# Production of Edible Films Based on Pea Starch with Incorporation of Active Compounds Obtained from the Purple Araçá (*Psidium myrtoides*)

**DOI:** 10.3390/polym13183134

**Published:** 2021-09-16

**Authors:** Thainá Stéphanie Martins de Freitas, Vitor Augusto dos Santos Garcia, Cristina Tostes Filgueiras, José Ignacio Velasco, Farayde Matta Fakhouri

**Affiliations:** 1 Faculty of Engineering, Federal University of Grande Dourados, Dourados 79804-970, Brazil; thaina.smf@gmail.com (T.S.M.d.F.); garcia.vitoraugusto@usp.br (V.A.d.S.G.); cristinafilgueiras@ufgd.edu.br (C.T.F.); 2 Faculty of Animal Science and Food Engineering, University of São Paulo, Pirassununga 13635-900, Brazil; 3 Poly2 Group, Department of Materials Science and Engineering, Universitat Politècnica de Catalunya (UPC BarcelonaTech), Carrer Colon 11, 08222 Terrassa, Spain; jose.ignacio.velasco@upc.edu

**Keywords:** anthocyanins, antimicrobial activity, flavonoids, packaging, phenolic compounds

## Abstract

The aim of this study was to incorporate the active compounds present in purple araçá (*Psidium myrtoides*) in pea starch-based films and to verify the influence of different plasticizers (glycerol, sorbitol, and polyethylene glycol 400) on film properties. Films were produced and characterized in relation to visual appearance, active compounds, antimicrobial activity, and mechanical and barrier properties. Pea starch has a high amylose content and a final viscosity of 5371.5 RVU, which contributes to the elaboration of films even without the addition of plasticizers. Purple araçá and pea starch formed films with good water vapor barrier characteristics (0.398 g·mm/m^2^·h·KPa) and low solubility (33.30%). Among plasticizers, sorbitol promoted a lower permeability to water vapor. The selected formulations, 0%, 20%, and 30% sorbitol, presented a high concentration of phenolic compounds (1194.55, 1115.47, and 1042.10 mg GAE 100 g^−1^, respectively) and were able to inhibit the growth of *Staphylococcus aureus*. Therefore, films contained the active compounds of purple araçá and potential to be used as food packaging.

## 1. Introduction

The production of films with biodegradable polymers such as polysaccharides, lipids, and proteins is being studied to be used as an alternative to minimize the use of petroleum-derived plastics [1,2,3,4,5]. Among the natural starch sources for film production, we can highlight peas, of which the extracted starch has an amylose content of 35 to 65% [6], favoring film production [7]. However, according to Fonseca et al. [3], starch films are brittle and more rigid; therefore, it is necessary to use plasticizers to improve these properties.

Plasticizers efficiency varies with the concentration and type of polymer added to films [3]. Plasticizers commonly used in the production of films are polyols, such as glycerol, sorbitol, and polyethylene glycol [8]. Otoni et al. [9] and Azeredo et al. [10] produced a film with mashed papaya and pomegranate juice, respectively, and identified that sugars present in fruits provided a plasticizing effect, as they reduced the stiffness and increased the extensibility of pectin films.

In addition to biodegradable films being produced from natural polymers, they can be edible with the possibility of incorporating fruit and vegetable purees, pulp, juice, or extract at the time of preparation. These films can be considered a source of active compounds, showing antioxidant or antimicrobial activity [1]. Rodrigues et al. [11] produced biodegradable films based on mangarite starch and reported that the incorporation of copaiba oil attributed an antimicrobial activity to films. Fakhouri et al. [12] produced films rich in ascorbic acid with the incorporation of cranberry in the arrowroot starch polymer matrix. Different studies in the literature report the production of edible packaging materials from different sources of starch and natural compounds, such as blackberry [13], guabiroba pulp [14], and acerola pulp [15]. In addition to compounds obtained from fruits, other bioactive substances based on natural ingredients have been used for obtaining antimicrobial and antioxidant activity [16,17].

Purple araçá (*Psidium myrtoides*) is a fruit with a fleshy and sweet pulp [18] and during maturation it presents a color change between green, orange, carmine, and, when ripe, atropurpure [19]. According to several studies in the literature, purple araçá has a high concentration of anthocyanins [20,21,22,23]. Thus, the addition of fruit pulp in the polymer matrix can contribute to increase the content of active compounds in films, as well as nutritional properties, in addition to altering films’ visual aspects and properties.

Given the above, the objective of this study was to develop biodegradable films incorporating active compounds present in the pulp and bark of purple araçá and to investigate the effect of different polyol plasticizers (glycerol, sorbitol, and polyethylene glycol 400) on the properties of pea starch-based films. Pea starch was characterized in relation to microstructure, amylose content, and paste properties. The films were distinguished in relation to visual appearance, thickness, color parameters, contact angle, water vapor permeability, solubility in water, mechanical properties, total phenolic compounds content, and antioxidant activity.

## 2. Materials and Methods

### 2.1. Materials

Purple araçá (*Psidium myrtoides*) was acquired in the city of Dourados, Mato Grosso do Sul (latitude of 22°06′01.5′′ S and longitude of 54°43′04.7′′ W). For the film production, pea starch (Romariz, São Paulo, Brazil), glycerol (Vetec, Rio de Janeiro, Brazil), sorbitol (Dinâmico, São Paulo, Brazil), and polyethylene glycol 400 (Neon, São Paulo, Brazil) were used. For the analysis of active compounds, ethylic (Neon, 95%, Suzano, Brazil), Folin-Ciocalteau (Dinamica, Indaiatuba, Brazil), sodium carbonate (Impex São Paulo, Brazil), gallic acid (Dinâmica, Santa Catarina, Brazil), sodium acetate (Proquímicos, Rio de Janeiro, Brazil), potassium chloride (Synth, Sao Paulo, Brazil), hydrochloric acid (Vetec, Duque de Caxias, Brazil), aluminum trichloride (Synth, São Paulo, Brazil), and quercitin (Sigma-Aldrich, São Paulo, Brazil). For microbiological analysis, bacterial strains of *Staphylococcus aureus* (ATCC 25923) and *Escherichia coli* (ATCC WDCM00013), Brain Heart Infusion (BHI) broth (Prodimol Biotecnologia, Belo Horizonte, Brazil), and Mueller–Hinton agar (Kasvi, Paraná, Brazil) were used.

### 2.2. Pea Starch Characterization

#### 2.2.1. Starch Granule Microstructure

The microstructure of starch granules was evaluated using an Optical Microscope (DMLM-Leica, Cambridge, UK). Granules were dispersed in glycerol and observed in transmitted light mode with 200 times magnification with and without polarized light.

#### 2.2.2. Amylose Content

Amylose content was determined by the colorimetric method, as described by Da Silva et al. [24]. Amylose PA (Sigma-Aldrich, MO, USA) was used as a standard. The amylose determination was performed in triplicate.

#### 2.2.3. Paste Properties (RVA)

Paste properties were determined in a Rapid Visco Analyzer (RVA) viscometer. Samples were suspended in 13% (b.s.) starch and heated between 25 to 95 °C observing an elevation at 6 °C/min and then cooled to 50 °C. Suspension stirring was maintained at 960 rpm for 10 sec, then 160 rpm for the remainder of the test time. Viscosity was expressed in RVU (Rapid Visco Unit) units. The paste properties determination was performed in triplicate.

### 2.3. Production of Pea Starch Films

Films were produced by the casting technique, using purple araçá peel and pulp solution obtained according to Freitas et al. [23]; pea starch (4%); and as plasticizers glycerol, sorbitol, and polyethylene glycol 400 at the concentrations of 0%, 20%, and 30% in relation to the starch mass. From the araça, only the seed was removed, and the pulp and peel were used in an integral way in the elaboration of the films. Initially, the starch and the plasticizer were dispersed separately into the araçá extract at room temperature and under manual stirring for approximately 3 min, after the dispersion formation, solutions were solubilized at 85 °C for 5 min in a thermostatic bath (Fisatom 550, Sao Paulo, Brazil) under manual stirring. Then, 25 mL of the filmogenic solution was dispersed in PS petri dishes (140 × 15 mm) and kept at room temperature for 48 h for drying.

### 2.4. Characterization of Edible Films

#### 2.4.1. Visual Appearance and Thickness

Edible films were evaluated for their visual and tactile characteristics as described by Nogueira, Fakhouri, and de Oliveira [25] for homogeneity (insoluble particles), continuity (absence of cracks), and flexibility (ease of removal from the dish). Films average thickness was determined with the aid of a digital micrometer (Mitutoyo, Japan), taking 15 random measurements.

#### 2.4.2. Color Parameters

The evaluation of film color parameters (Luminosity, chroma a* and chroma b*) was performed with the aid of a colorimeter (Konica Minolta CR-400, NJ, USA), performing 5 random measurements on the edible film with an area of 15.39 mm^2^. The color parameters determination was performed in triplicate per formulation (totaling 9 samples).

#### 2.4.3. Contact Angle

The determination of edible films contact angle was performed using an Attension Theta Lite tensiometer (KSV Instrument, Helsinki, Finland). Samples of edible films (3 × 2 cm) were fixed on the base of the equipment and with the aid of a microsyringe (Hamilton, Reno, USA) a drop of deionized water (5 µL) was placed on films surface. After 10 s, images of purple araçá films were recorded using the equipment’s digital camera. Subsequently, the contact angle was determined by analyzing the images using the Attension Theta Lite software (Version 4.1.9.8, Helsinki, Finland). The contact angle determination was performed in triplicate per formulation (totaling 9 samples).

#### 2.4.4. Water Vapor Permeability (WVP)

WVP was determined by ASTM E-96 [26] standard method. Initially, films were cut into 5 cm diameter discs and preconditioned at a temperature of 25 °C (relative humidity of 55% MgNO_3_) for 48 h. Then, discs were placed in acrylic cells with calcium chloride inside (25 °C) and were stored in desiccators with a saturated sodium chloride solution (75% relative humidity at 25 °C). Twelve weighings were carried out over 29 h, six weighings every 1 h in the first hours and another six in the last hours, to control the calcium chloride mass gain in order to determine how permeable the film was to water vapor. The WVP determination was performed in triplicate per formulation (totaling 9 samples).

(1) $$WVP=\left(\frac{e}{A\Delta P}\right)\times M$$

where *WVP* = water vapor permeability (g·mm·m^−2^·day^−1^·kPa^−1^); *e* = average film thickness (mm); *A* = permeation area (m^2^); ∆*P* = partial difference in vapor pressure between the two sides of films (kPa, at 25 °C); and *M*= absorbed moisture rate.

#### 2.4.5. Solubility in Water

Solubility in water was determined according to Gontard, Guilbert, and Cuq [27]. Samples (2 cm in diameter) were dried for 24 h at 105 °C in an oven with air circulation (Lucadema, Luca-80/100) to determine the initial dry mass, then they were placed in 50 mL of distilled water and submitted to slow stirring (75 rpm) in an incubator with orbital stirring (Lucadema, Luca-223) for 24 h and at room temperature. Non-solubilized samples were dried at 105 °C for another 24 h to determine the final dry mass. The solubility was determined according to Equation (2) [25]:

(2) $$Solubilized\;Material=\left(\left(m_{si}-m_{f}\right)/m_{si}\right)\times 100$$

where (*m_si_*) is the initial dry mass of films and (*m_sf_*) is the final dry mass of non-solubilized films.

#### 2.4.6. Mechanical Properties

Tensile strength and elongation were determined according to ASTM D 882-83 [28], with modifications, in a texturometer (TextureAnalyser, TA.XTplus, Godalming, UK). Edible film samples (70 mm long and 25 mm wide) and initial claw separation of 50 mm and speed of 1 mm/s. To determine the tensile strength, the maximum force was divided by the film sectional area (film width × thickness). Elongation was calculated by dividing extension values by the initial claw separation (50 mm) and multiplying by 100. The mechanical properties determination was performed in quintuplicate per formulation (totaling 15 samples).

### 2.5. Content of Total Phenolic Compounds and Antioxidant Activity

#### 2.5.1. Phenolic Compounds

The content of phenolic compounds was determined by the Folin–Ciocalteu method [29]. Samples (0.5 mL) together with 2.5 mL of Folin–Ciocalteu reagent were placed in tubes that remained at rest protected from light for 5 min. Subsequently, 2 mL of anhydrous sodium carbonate solution (7.5%) was added to the tube, homogenized, and kept in the absence of light for 2 h. Soon after, the absorbance was determined in a spectrophotometer (Jenway 7310 Staffordshire, UK) at 740 nm. The calibration curve was prepared using gallic acid (0.01672 to 0.07524 mg/mL) and total phenolic compounds was expressed in mg of gallic acid equivalent (EAG) 100 g^−1^. (y = 12.408x + 0.0171, R^2^ = 0.993).

#### 2.5.2. Flavonoids

To determine the flavonoid content, samples (500 µL) were solubilized together with 1.5 mL of 95% ethanol, 100 µL of aluminum chloride (10%), 100 µL of sodium acetate (1 mol L^−1^), and 2.8 mL of distilled water according to Lin and Tang [30]. After 40 min, the absorbance was determined at 415 nm in a spectrophotometer. The calibration curve was prepared with quercetin (0.01 to 0.16 mg/mL) and results expressed in mg quercetin equivalent (QE) per 100 g of sample. (y = 5417.6x + 0.0178, R^2^ = 0.999)

#### 2.5.3. Anthocyanins

The concentration of anthocyanins was determined using the differential pH method described by Lee, Durst, and Wroslstad [31]. Edible film samples (1 mL) were solubilized in 4 mL of buffer solutions pH 1.0 (0.025 mol L^−1^) and pH 4.5 (0.4 mol L^−1^) sodium acetate buffer solutions, respectively. Solutions were homogenized and the absorbance determined in a spectrophotometer (Jenway 7310, Staffordshire, UK) at 520 and 700 nm. The anthocyanin content was calculated as cyanidin-3-glucoside equivalent using Equations (3) and (4), and the results were expressed in mg of cyanidin-3-glucoside equivalent (C3G) per 100 g of sample.

(3) $$Anthocyanins=\left(AP_{M}F_{D}10^{3}V/\varepsilon Lm\right)\times 100$$

(4) $$A=\left[{\left(A_{520}-A_{700}\right)}_{pH1.0}\right]-\left[{\left(A_{520}-A_{700}\right)}_{pH4.5}\right]$$

where *A* = difference between absorbances at pH 1.0 and 4.5; *P_M_* = molecular weight of cyanidin-3-glycoside (449.2 g mol^−1^); *F_D_* = Dilution factor; *V* = extract volume (L); 103 = g to mg conversion; *Ɛ* = cyanidin-3-glycoside molar extinction coefficient (26,900 L mol^−1^ cm^−1^); *L* = cell length (1 cm); *m* = sample mass (g).

### 2.6. Antimicrobial Activity

The antimicrobial activity of edible films was evaluated against *S. aureus* (Gram-positive) and *E. coli* (Gram-negative). Initially, the bacteria were cultivated in BHI medium at 35 °C for 18 h. Then, films were cut into discs (5.5 mm) and placed on Mueller–Hinton agar, which was seeded with 100 µL of *S. aureus* and *E. coli* cultures, separately. Samples were incubated at 35 °C for 24 h. The inhibition halo was determined using a ruler and results were expressed in mm.

### 2.7. Statistical Analysis

The InfoStat^®^ software (version 2018d, Buenos Aires, Argentina) was used to calculate the analysis of variance (ANOVA). The Tukey test was performed to determine the difference between means at a 95% confidence level.

## 3. Results and Discussion

### 3.1. Pea Starch Microstructure, Amylose Content, and Paste Properties

**Figure 1:**
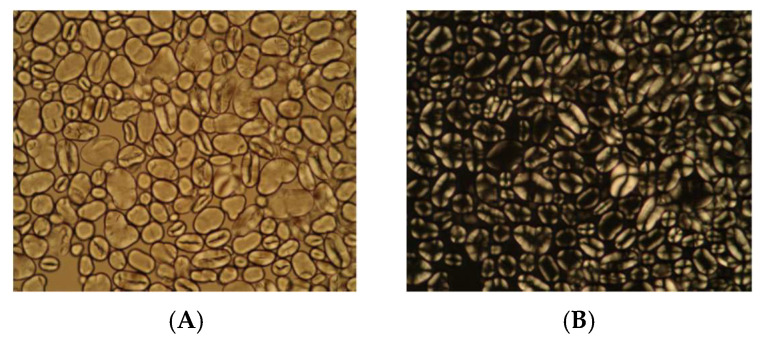
(**A**) Pea starch under non-polarized light (400× magnification) and (**B**) pea starch under polarized light (400× magnification).

Pea starch morphology was characterized by optical microscopy analysis and can be seen in Figure 1.

Analyses show that pea starch presents oval granules (Figure 1A), with a smooth and rounded surface. The malt cross, characteristic in starch granules under polarized light, is eccentric for pea starch (Figure 1B).

Pea starch had an amylose content of 44.45 ± 0.64%, a very high content that confirms the ability of this starch to form films, as the higher the amylose content, the greater the starch tendency to form films. This content is higher than those found by Nogueira et al. [32] arrowroot (*Maranta arundinaceae* L.) starch (35%). It is much superior to those found for white (16.61%), red (25.75%), and black (20.02%) rice starch [24] and also superior to the mangarite *Xanthosoma mafaffa* Schott starch (25.78%) [11].

**Figure 2:**
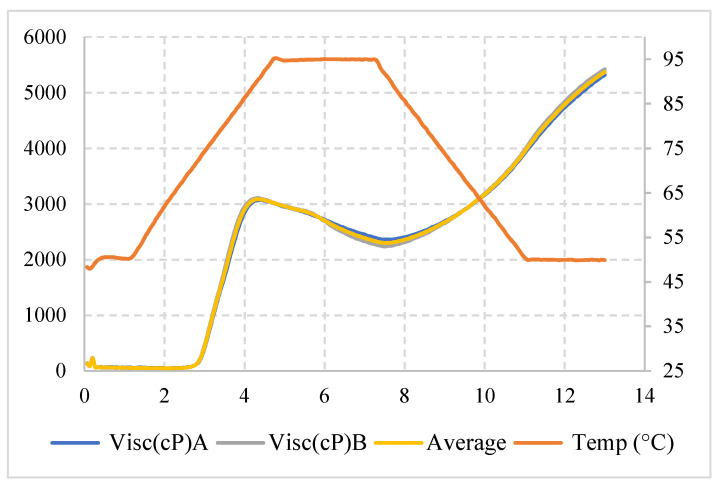
Rapid Visco Analyzer of pea starch.

In RVA (Figure 2), during the initial heating phase of an aqueous starch suspension, an increase in viscosity is recorded at a temperature of approximately 71.8 °C, a phase in which granules begin to swell. At this point, low molecular weight polymers, particularly amylose molecules, begin to leach out of granules. A viscosity peak of 67.5 RVU is obtained during pasting, a stage in which there are most fully swollen granules, intact granules, and the molecular alignment of any solubilized polymer has not yet occurred within the instrument friction field [33]. During the constant temperature phase granules begin to break down and polymer solubilization continues. At this point, there was a break in viscosity followed by a decrease in viscosity, which was approximately 1690 RVU. During the cooling phase, solubilized amylose polymers and some amylopectin branches begin to reassociate and another 3006 RVU increase in viscosity is recorded. This second increase in viscosity is known as a set-back tendency. At the end of the process, a final viscosity of 5371.5 RVU was observed, indicating the ability of this starch to form continuous and cohesive matrices.

Thus, pea starch has greater stability than cassava starch, as, according to Leonel and Cereda [34], the more abrupt the drop after the paste peak, the lower its stability at high temperatures and mechanical agitation. In addition, pea starch has a better film production characteristic, as, according to Rosenthal et al. [35], the higher the paste temperature, the higher the degree of association in amorphous zones of granules.

The pea starch viscosity analysis shows a relatively high final viscosity; this phenomenon may be due to the high amylose content present in this starch. Pea starch also shows stability to heating, due to less breakdown of starch granules after the maximum viscosity peak (Figure 2).

### 3.2. Film Characterization

#### 3.2.1. Visual Appearance and Color Parameters

**Figure 3:**
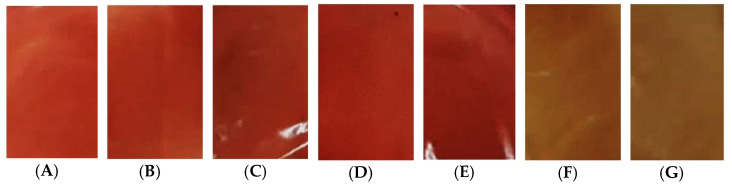
Edible films based on pea starch with incorporation of active compounds obtained from purple araçá: (**A**) 0%, (**B**) 20% GLI, (**C**) 30% GLI, (**D**) 20% SOR, (**E**) 30% SOR, (**F**) 20% PEG, and (**G**) 30% PEG.

Films produced with purple araçá peel and pulp, pea starch, without plasticizer and plasticized with glycerol (GLI), sorbitol (SOR) and polyethylene glycol 400 (PEG) at concentrations of 0%, 20%, and 30%, were easily removed from the support, did not show insoluble particles and cracks. Films’ visual appearance was not affected by different concentrations of plasticizer; however, films plasticized with polyethylene glycol 400 showed a difference in color compared to the others (Figure 3), possibly due to interactions in films’ polymer matrix, which during drying may have caused the matrix to darken.

The red color of the plasticizer-free film (control), with glycerol and sorbitol, was promoted by the purple araçá peel and pulp added to the film; however, the addition of polyethylene glycol caused a change in color making the film yellow, as shown in Figure 3 F,G, which can be confirmed by color parameters shown in Table 1. Compared to the control film (0%), the addition of polyethylene glycol significantly increased (*p* < 0.05) L* and b* values and decreased the a* value, indicating the tendency to yellow. Saberi et al. [36] produced films with pea starch, guar gum, and different types of plasticizer and identified that when adding polyethylene glycol 400, the formed film was more opaque and white than the others, they suggested that this fact could be related to the high molecular weight and the reduced amount of hydroxyl groups in this plasticizer. Moreover, the same was also observed by Laohakunjit and Noomhorm [8] and Razavi, Amini, and Zahedi [37] who produced films with rice starch and sage gum, respectively.

When evaluating each group separately (films plasticized with GLI, SOR, and PEG), the increase in the plasticizer concentration did not cause a significant difference between the color parameters evaluated, the only exception was observed for PEG, where the increase in the plasticizer concentration promoted a significant increase in chroma b*.

#### 3.2.2. Contact Angle, Water Vapor Permeability and Water Solubility

The contact angle data, water vapor permeability (WVP), and solubility of pea starch-based edible films with incorporation of active compounds obtained from purple araçá pulp can be observed in Table 2.

The hydrophobicity of edible films was evaluated by measuring the contact angle between the water and films surface. The incorporation of polyethylene glycol 400 at a higher concentration in the film reduced the contact angle value (Table 2), indicating an increase in hydrophilic characteristics of the polymer matrix. However, the lower concentration of this and the addition of other plasticizers (GLI and SOR) to the film did not promote a significant reduction (*p* < 0.05) in contact angle values, compared to the control (0%) and presented higher values than 65° suggesting that the surface of these films were more hydrophobic [38]. The higher concentration of polyethylene glycol 400 probably did not allow interaction with the polymeric matrix, thus making the hydroxyl groups available and providing a film with a more hydrophilic surface.

The water vapor permeability of food packaging must be reduced to provide as little moisture transfer as possible between the product and the surrounding environment [39]. The water vapor permeability of films increased significantly (*p* < 0.05) with incorporation of glycerol and polyethylene glycol 400, when compared to the control film (0%). However, sorbitol did not show statistical difference with the film without plasticizer.

The greater permeability to water vapor observed for edible films plasticized with glycerol possibly occurred because films had more hygroscopic characteristics than the other plasticizers (>contact angle), thus having greater interaction with water and polymeric matrix components [8]. Polyphenols present in purple araçá peel and pulp may have contributed to the lower water vapor permeability of films. Feng et al. [40] elaborated starch films incorporating tea polyphenols and suggested that the low PVA value obtained in the research occurred due to the formation of hydrogen bonds between starch and polyphenols, which reduced the availability of hydrogen groups to bind to water molecules.

Edible films without plasticizer (control) had a lower solubility in water when compared to films with plasticizers used in this study (33.30%). Films solubility increased as the number of hydroxyl groups in plasticizers increased. The film plasticized with sorbitol was more soluble, followed by films plasticized with glycerol and polyethylene glycol 400. Plasticizers provided greater affinity of the polymer to water, as they reduce the interaction of polymer molecules with each other and make them more soluble because they are hydrophilic [41].

Determining the water solubility of films produced with biopolymers is important, as it defines their resistance or tolerance to water and enables the identification of the best options for application [42]. Films with low solubility are desirable for product storage [8]; however, more soluble edible films are feasible to coat foods where they can be consumed along with the packaged product.

#### 3.2.3. Mechanical Properties

Edible films had thickness values between 0.158 and 0.171 mm (Table 3). The control film (0%) showed statistical difference only to the 30% PEG film, which had greater thickness; this result may be related to the plasticizer higher molecular weight. According to Razavi, Amini, and Zahedi [37], plasticizers can produce films with greater thickness, as they break the intermolecular bonds between polymer chains, thus restructuring them and forming an expanded structure with greater volume.

The mechanical properties (tensile strength and elongation) of edible films with different plasticizers are shown in Table 3. The addition of plasticizer significantly affected (*p* < 0.05) the mechanical properties of films. Films with plasticizers had lower tensile strength values than the control (0%), with the exception of the 20% PEG formulation.

The incorporation of 30% glycerol and polyethylene glycol promoted less elongation to the film, while sorbitol caused greater elongation and did not differ significantly from the control (0% P). The greater number of sorbitol hydroxyl groups may have influenced this result, as plasticizers can reduce the strength and increase the elasticity of films by promoting greater mobility of the polymer structure chains, due to the hydrogen bonds between them [37].

### 3.3. Total Phenolic Compounds Content and Antioxidant Activity

Based on previous results, in addition to the control film (0%), films plasticized with 20% and 30% sorbitol were selected as the best formulations for application, these were chosen due to their lower water vapor permeability (0.467 and 0.477 g·mm/m^2^·h·KPa, respectively), high solubility (40.69 and 43.63%, respectively), and good mechanical properties compared to the other formulations analyzed.

The lowest WVP of the film is required, because when applying it as a packaging or as an edible coating, it promotes better food protection against moisture. High solubility is desired so that we can take advantage of all components of purple araçá peel and pulp that can be inserted in this film, as active and antimicrobial compounds. Therefore, these films were also characterized for phenolic compounds, flavonoids, anthocyanins, and antimicrobial activity.

Table 4 presents the concentration of total phenolic compounds, flavonoids, and anthocyanins of pea-based edible films with incorporation of active compounds obtained from purple araçá and different plasticizers. Films incorporated with active compounds are of great interest to food industries due to better preservation of packaged food quality [43].

Through results of total phenolic compounds (Table 4), it was possible to identify that active compounds of purple araçá were incorporated in edible films and despite the process applied, components were largely preserved, as the purple araçá had a content of phenolic compounds of 2347.23 and 2200.23 mg GAE per 100 g for peel and pulp, respectively [23]. The content of phenolic compounds significantly reduced (*p* < 0.05) with the incorporation and increase in the plasticizer concentration, possibly due to the increase in plasticizer ratio in relation to the fruit ratio in the mixture.

The content of phenolic compounds indicates the great potential of these films for application as food packaging because they contain active compounds that help preserve products during storage. The application of films for this purpose has already been studied by some researchers such as Reis et al. [43] who incorporated active compounds in cassava starch films through mango pulp and yerba mate extract and identified that phenolic compounds and flavonoids present in these were efficient in preserving palm oil when films were tested as packaging for this product due to the antioxidant action of these compounds.

The flavonoid content did not differ significantly (*p* < 0.05) between formulations, indicating that sorbitol incorporation did not interfere with the preservation of this compound in the film. The incorporation of flavonoids in the film is important because these are natural compounds with antioxidant capacity that can provide health benefits and can also be used to package and preserve food.

Through Table 4 we can identify that regardless of the plasticizer addition, anthocyanins were incorporated into the film and attributed the red color to them. Anthocyanins are susceptible to oxidation when exposed to light and oxygen, but when incorporated into the film matrix, they can act as a barrier to factors that cause oxidation [44], thus preserving these and promoting their availability for consumption. Anthocyanins can also be incorporated into films to serve as food quality indicators because their color changes in the presence of different pH values [45]. Silva-Pereira et al. [46] produced films incorporated with anthocyanins obtained from red cabbage extract to evaluate films’ efficiency as pH indicators in fish quality evaluation. According to Garcia et al. [47], different concentrations of macromolecules can provide different protections to the active components, depending on the intrinsic characteristics of each molecule.

### 3.4. Antimicrobial Activity

**Figure 4:**
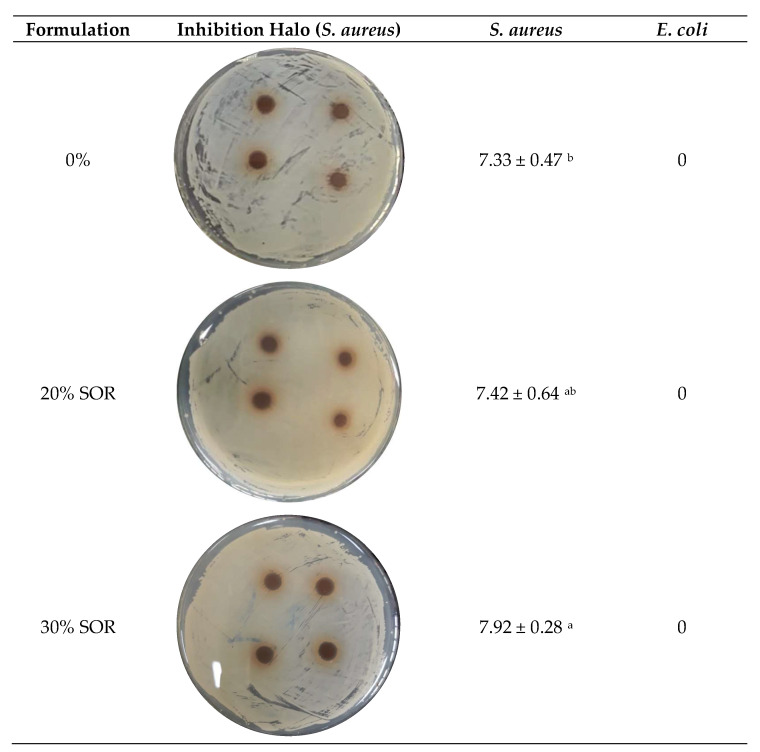
Inhibition halo test for *S. aureus* and antimicrobial activity against *S. aureus* and *E. coli* of pea starch-based edible films with incorporation of active compounds obtained from purple araçá and sorbitol. Different letters in the same column indicate significant difference (*p* < 0.05).

When performing halo inhibition tests to determine the antimicrobial activity of edible films, regardless of added plasticizer concentration (0%, 20%, and 30%), it was identified that films were able to inhibit the growth of *S. aureus*; however, they were not able to inhibit *E. coli* (Figure 4). *S. aureus* is more sensitive to polyphenols than E. *coli* [40]; this fact may be related to the hydrophilic membrane present in Gram-negative bacteria that hinder the passage of hydrophobic compounds [9]. The visualization of *S. aureus* growth inhibition zone formed around the film discs can be exemplified in Figure 4.

By adding the plasticizer to the film, it significantly favored *S. aureus* inhibition, especially at the highest concentration (30% sorbitol), which may have occurred due to the formation of a more homogeneous matrix, due to the greater interaction between the plasticizer and the polymer matrix (Figure 4).

## 4. Conclusions

It was possible to obtain edible films from the pulp and peel of purple araçá with pea starch. Pea starch has a high amylose content and a final viscosity of 5371.5 RVU, which favors its use in the preparation of edible films. Purple araçá provided films with a bright red color, with the exception of films plasticized with polyethylene glycol 400, which presented higher L* and b* values and lower chroma a* values than the others. Produced films showed low tensile strength and films elongation ranged between 32.75% (for films plasticized with polyethylene glycol) and 48.08% (films plasticized with sorbitol). Films produced with 20 and 30% sorbitol had lower water vapor permeability values and higher water solubility values. The control formulations and those plasticized with sorbitol presented active compounds and enabled the inhibition of *Staphylococcus aureus*. Therefore, films produced with purple araçá proved to be a good alternative for the production of packaging with incorporation of active compounds.

## Figures and Tables

**Table 1:** Color parameters (L*, chroma a* and chroma b*) of starch-based edible films with incorporation of active compounds obtained from purple araçá and different plasticizers, being: glycerol (GLI), sorbitol (SOR) and polyethylene glycol (PEG).

Formulations	Color Parameters		
	L*	a*	b*
0%	53.11 ± 2.99 ^bc^	35.92 ± 3.93 ^a^	28.72 ± 1.84 ^d^
20% GLI	53.86 ± 3.04 ^bc^	34.25 ± 2.75 ^ab^	29.87 ± 1.30 ^c^
30% GLI	54.83 ± 2.44 ^b^	33.21 ± 2.62 ^b^	29.39 ± 1.91 ^cd^
20% SOR	53.59 ± 2.08 ^bc^	34.74 ± 1.85 ^ab^	28.72 ± 1.03 ^d^
30% SOR	52.89 ± 2.65 ^c^	35.34 ± 1.82 ^a^	29.21 ± 1.22 ^cd^
20% PEG	61.84 ± 2.62 ^a^	17.94 ± 4.22 ^c^	34.45 ± 1.57 ^b^
30% PEG	60.61 ± 2.08 ^a^	17.46 ± 1.72 ^c^	35.72 ± 1.31 ^a^

Different letters in the same column indicate significant difference (*p* < 0.05).

**Table 2:** Contact angle, water vapor permeability (WVP), and solubility of pea starch-based edible films with incorporation of active compounds obtained from purple araçá and different plasticizers, being: glycerol (GLI), sorbitol (SOR), and polyethylene glycol (PEG).

Formulations	Contact Angle (°)	WVP (g.mm/m^2^.h.KPa)	Solubility (%)
0%	76.77 ± 2.37 ^a^	0.398 ± 0.043 ^e^	33.30 ± 4.94 ^d^
20% GLI	66.44 ± 12.42 ^a^	0.610 ± 0.058 ^ab^	40.78 ± 2.34 ^abc^
30% GLI	78.05 ± 2.52 ^a^	0.669 ± 0.076 ^a^	41.92 ± 1.11 ^ab^
20% SOR	67.95 ± 1.58 ^a^	0.467 ± 0.020 ^de^	40.69 ± 0.65 ^abc^
30% SOR	76.60 ± 5.10 ^a^	0.477 ± 0.022 ^de^	43.63 ± 0.49 ^a^
20% PEG	67.96 ± 7.77 ^a^	0.507 ± 0.034 ^cd^	37.71 ± 2.11 ^c^
30% PEG	49.96 ± 4.20 ^b^	0.574 ± 0.030 ^bc^	38.18 ± 1.41 ^bc^

Different letters in the same column indicate significant difference (*p* < 0.05).

**Table 3:** Thickness, tensile strength (TR), and elongation of pea starch-based edible films with incorporation of active compounds obtained from purple araçá and different plasticizers, being glycerol (GLI), sorbitol (SOR), and polyethylene glycol (PEG).

Formulations	Thickness (mm)	TR (MPa)	Elongation (%)
0%	0.158 ± 0.017 ^b^	3.28 ± 0.21 ^a^	45.87 ± 5.06 ^ab^
20% GLI	0.160 ± 0.020 ^b^	2.31 ± 0.23 ^b^	42.25 ± 5.58 ^bc^
30% GLI	0.160 ± 0.018 ^b^	1.83 ± 0.12 ^c^	40.88 ± 4.34 ^c^
20% SOR	0.163 ± 0.020 ^ab^	2.46 ± 0.37 ^b^	43.43 ± 5.33 ^abc^
30% SOR	0.163 ± 0.019 ^ab^	2.39 ± 0.25 ^b^	48.08 ± 3.66 ^a^
20% PEG	0.161 ± 0.021 ^b^	3.17 ± 0.41 ^a^	46.44 ± 4.79 ^ab^
30% PEG	0.171 ± 0.018 ^a^	2.42 ± 0.28 ^b^	32.75 ± 4.16 ^d^

Different letters in the same column indicate significant difference (*p* < 0.05).

**Table 4:** Total phenolic compounds, flavonoids, and anthocyanins of edible films based on starch with incorporation of active compounds obtained from purple araçá and sorbitol (SOR).

Formulations	Phenolic Compounds ^1^	Flavonoids ^2^	Anthocyanins ^3^
0%	1194.55 ± 77.64 ^a^	48.88 ± 1.11 ^a^	31.72 ± 3.94 ^a^
20% SOR	1115.47 ± 67.89 ^b^	47.70 ± 2.85 ^a^	29.06 ± 3.45 ^a^
30% SOR	1042.10 ± 58.69 ^c^	46.91 ± 3.79 ^a^	28.95 ± 1.01 ^a^

^1^ mg gallic acid equivalent per 100 g; ^2^ mg quercitin equivalent per 100 g; ^3^ mg cyanidin 3-glycoside per 100 g; Different letters in the same column indicate a significant difference (*p* < 0.05).
